# 4D flow of the whole heart and great vessels using real time self respiratory gating

**DOI:** 10.1186/1532-429X-11-S1-P3

**Published:** 2009-01-28

**Authors:** Sergio A Uribe Arancibia, Philipp Beerbaum, Allan Rasmusson, Thomas Sangild Sørensen, Reza Razavi, Tobias Schaeffter

**Affiliations:** 1 King's College London, London, UK; 2 University of Aarhus, Aarhus, Denmark

**Keywords:** Stroke Volume, Free Breathing, Respiratory Gating, Quantify Blood Flow, Respiratory Gating Technique

## Objective

To evaluate the feasibility of a 4D-flow sequence of the whole heart and great vessel to retrospectively quantify blood flow within the entire heart.

## Background

4D-flow has been introduced as a means of acquiring anatomical and three-directional velocity information for all pixels within a 3D volume over different time points. The acquisition time of such data sets is long and respiratory compensation is thus required. However, navigator beams could disturb the steady state and are time consuming, which can be circumvented by using a self-navigation approach. Here, we present a new technique for the acquisition of 4D-flow data of an isotropic saggital volume using a real time self gating technique. The data can thereafter be reformatted in any clinical view allowing the quantification of flow in different vessels with arbitrary orientations. This is important in congenital heart (CH) patients where scan planning can prolong the overall scan-time.

## Methods

### Self navigation

A 3D Phase Contrast (PC) retrospective cardiac trigger sequence was used to acquire 4D flow data. The sequence was modified to enable the acquisition of an extra *k*_0_ profile at certain time intervals. These profiles were used to derive the breathing motion and to respiratory gate the acquisition in real-time. All the modifications were integrated into the software of a clinical MR-scanner (Philips Healthcare, Best).

### Experiments

4D-flow data of the whole heart and great vessels was obtained in 15 volunteers on a 3 T scanner (resolution of 2.5 mm^3^ and 25 cardiac phases, acceptance window = 8 [mm]). To study the reproducibility of the technique, two 4D-flow data sets were acquired with self respiratory gating. For comparison one 4D flow with two averages were obtained without respiratory gating. Furthermore, 2D PC scans were obtained at the level of the AO, PA, LPA and RPA. Statistical analysis and Bland-Altman plots were used to compare the stroke volume (SV) derived from the 4D and 2D flow acquisitions.

Furthermore a 4D-flow data set was obtained on a CH patient with a repaired coarctation and a levo-atrial cardinal vein (LACV) connecting the left atrium with the brachiocephalic vein.

## Results

**Figure 1:**
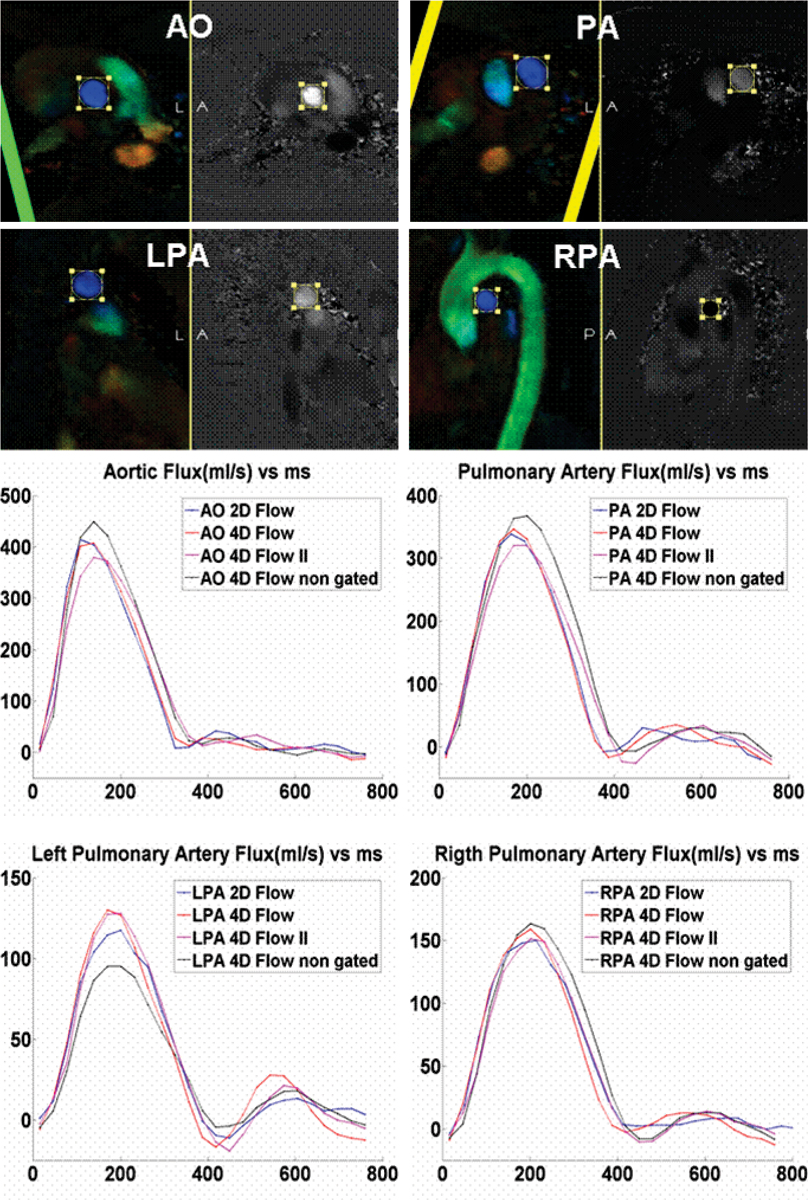
Figure 1

**Figure 2:**
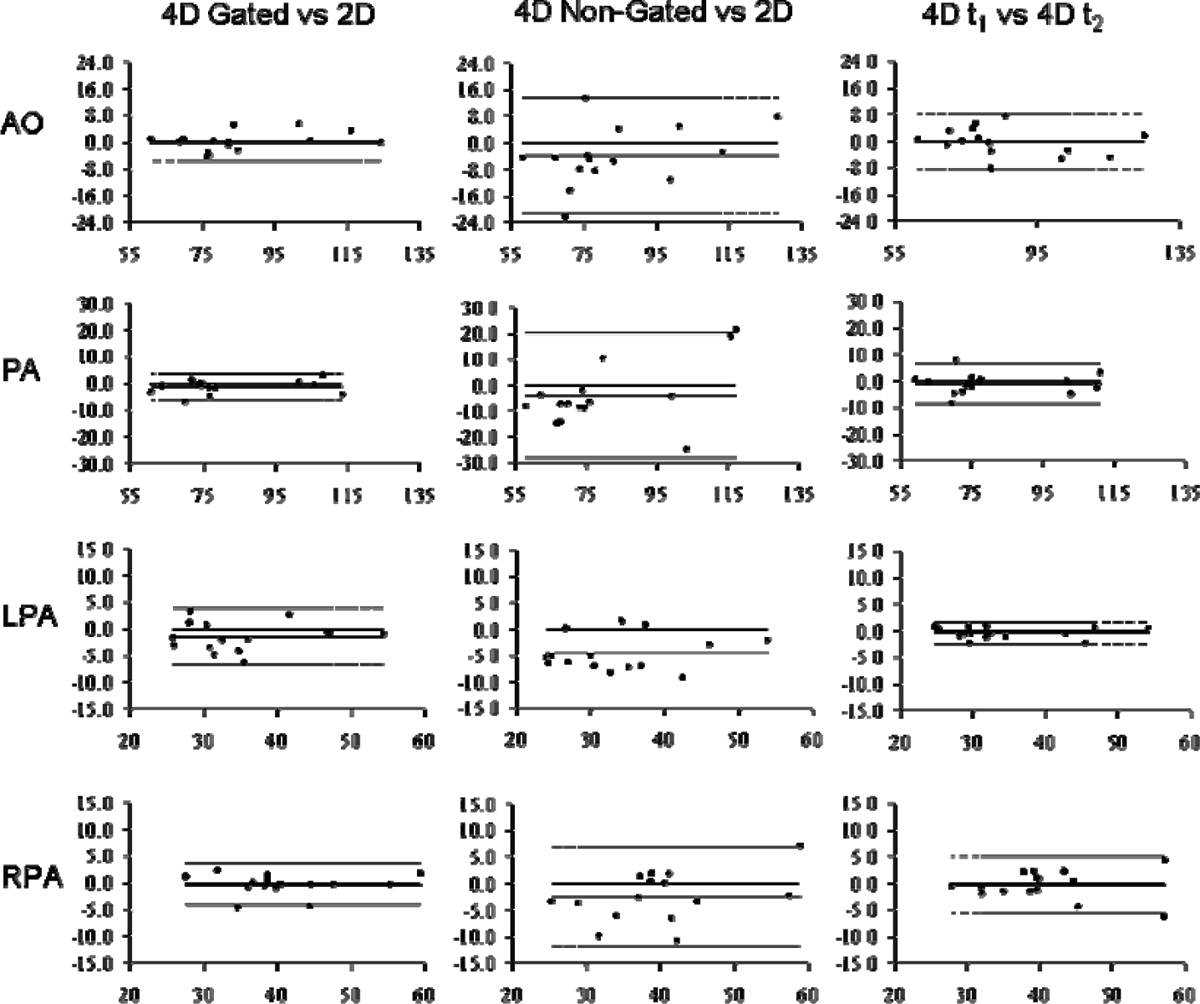
Figure 2

The self-respiratory gated acquisition resulted in an overall scan time of 15 ± 2.8 min. Figure 1 shows reformatted slices of the 4D-flow data and an example of the flux in one volunteer. Table 1 shows the SV comparison for the different techniques. No statistically difference was found between the different pairs of data. However, Bland-Altman plots showed a larger standard deviation and bias for the pair "4D non-gated to 2D" (Figure 2). Therefore the 4D-flow gated scan showed to be more accurate than the 4D-flow data obtained during free breathing.

**Table 1:** Stroke volume (mean and stdev) of the different acquisitions for all measured vessel

	4D gated t1Mean [ml] ± sdv [ml]	2DMean [ml] ± sdv [ml]	4D non gatedMean [ml] ± sdv [ml]	4D gated t2Mean [ml] ± sdv [ml]
AO	85.86 ± 18.54	85.65 ± 17.54	81.78 ± 21.03	85.62 ± 17.37
PA	81.38 ± 17.67	82.71 ± 17.07	78.65 ± 23.76	80.23 ± 17.71
LPA	34.93 ± 9.33	36.15 ± 9.14	31.19 ± 9.52	34.57 ± 9.27
RPA	40.74 ± 9.07	40.67 ± 8.90	38.05 ± 10.99	40.12 ± 8.91

**Figure 3:**
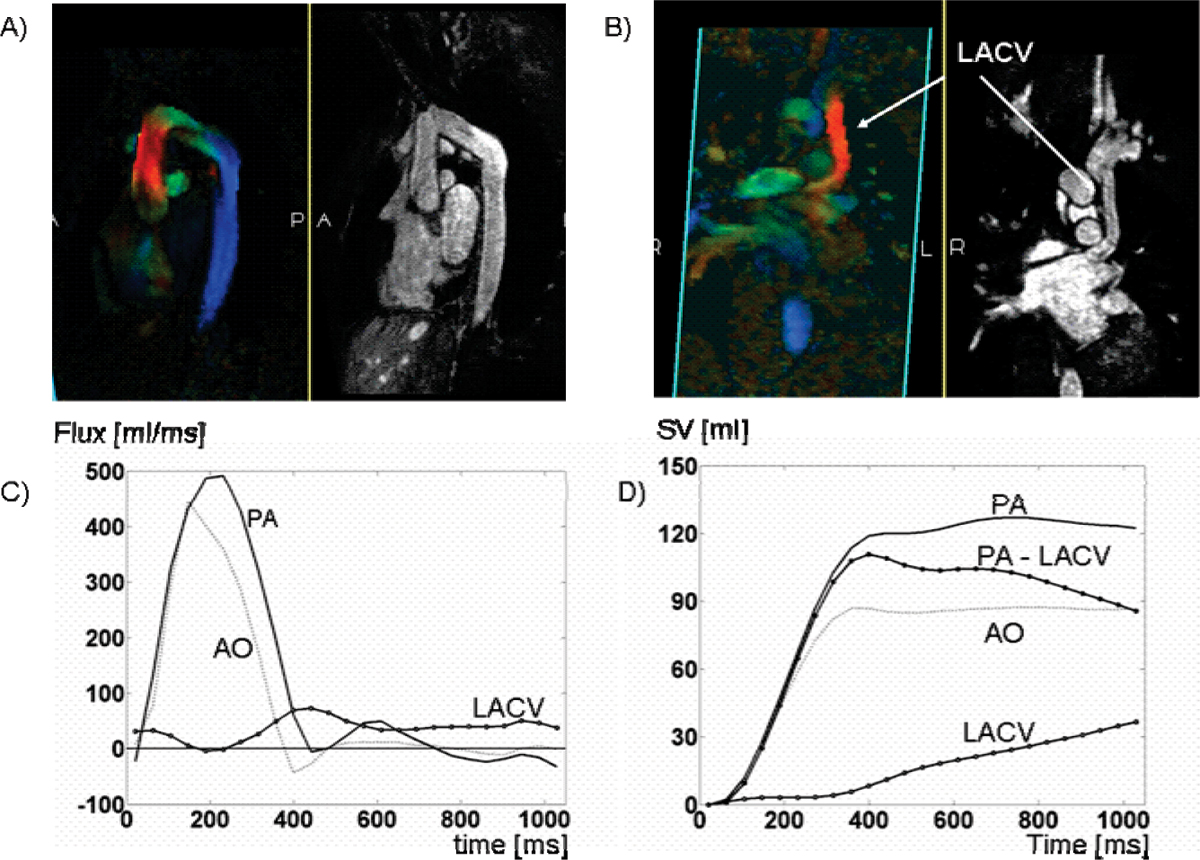
Figure 3

Figure 3 shows the data obtained in the congenital patient. This data set was used to visualize the flow pattern in the region of coarctation. Furthermore, it allows the accurate quantification of the flux in the AO, PA, and in the LACV (Figure 3C, D). We found the SV in the PA and AO was 127.91 [ml] and 90.68 [ml] respectively. The SV difference (37.22 [ml]) between the PA and AO was due to the connection caused by the LACV. Indeed the SV measured by the 4D flow in the LACV was 38.06 [ml], which matches the difference of the SV between the PA and AO.

## Conclusion

We have demonstrated the feasibility of 4D-flow on the whole heart using a self respiratory gating technique. The method allows retrospectively flow quantification within the entire heart and great vessel from data obtained in a single free breathing scan. This method represents a practical advance for an easier cardiac MR examination and showed to be very valuable in congenital patients.

